# Prospective association of dietary soy and fibre intake with puberty timing: a cohort study among Chinese children

**DOI:** 10.1186/s12916-022-02320-5

**Published:** 2022-04-04

**Authors:** Jingyuan Xiong, Yujie Xu, Xueting Liu, Xiaoyu Wang, Shufang Shan, M. James C. Crabbe, Li Zhao, He Fang, Guo Cheng

**Affiliations:** 1 West China School of Public Health and West China Fourth Hospital and Healthy Food Evaluation Research Center, Sichuan University, Chengdu, People’s Republic of China; 2 Laboratory of Molecular Translational Medicine, Center for Translational Medicine, Key Laboratory of Birth Defects and Related Diseases of Women and Children (Sichuan University), Ministry of Education, Department of Pediatrics, West China Second University Hospital, Sichuan University, Chengdu, Sichuan People’s Republic of China; 3 Wolfson College, Oxford University, Oxford, OX2 6UD UK; 4 Institute of Biomedical and Environmental Science & Technology, University of Bedfordshire, Luton, LU1 3JU UK

**Keywords:** Dietary soy, Dietary fibre, Puberty, equol, Cohort study

## Abstract

**Background:**

Dietary phytoestrogens have been suggested to influence puberty timing, a critical stage for well-being in adulthood. We hypothesized that childhood soy intake might prospectively influence puberty timing and that dietary fibre and the key isoflavone metabolite equol might play roles.

**Methods:**

Cox proportional hazard regression models were performed in 4781 children (2152 girls and 2629 boys) aged 6–8 years old from the Chinese Adolescent Cohort Study for whom a food frequency questionnaire at baseline and information about potential confounders were available. Anthropometry and pubertal status including age at Tanner stage 2 for breast development (B2) or age at the initiation of gonadal growth (G2), and age at menarche (M) or voice break (VB) were assessed annually. Equol excretion was determined by urine samples from 1311 participants.

**Results:**

Among girls and boys, higher soy intake was associated with later puberty timing (hazard ratio (HR)-B2: 0.88 (95% CI, 0.80–0.96), *p*=0.02; HR-M, 0.87 (0.77–0.94), *p*=0.01; HR-G2, 0.91 (0.82–0.98), *p*=0.013; HR-VB, 0.90 (0.82–0.9), *p*=0.02), independent of prepubertal body fatness and fibre intake. These associations were more pronounced among children with a high urinary equol level (*p*_for-interaction_ ≤ 0.04) or with a high cereal fibre intake (*p*_for-interaction_ ≤ 0.06). Intake of dietary fibre or its subtype was not prospectively associated with puberty onset after adjusting for dietary soy intake (*p*≥0.06).

**Conclusion:**

Higher childhood soy intake is prospectively associated with later puberty timing in both Chinese girls and boys, independent of prepubertal body fatness, and the association is particularly pronounced among individuals with a higher urinary equol level.

**Supplementary Information:**

The online version contains supplementary material available at 10.1186/s12916-022-02320-5.

## Background

Puberty is a critical period of time encompassing sequential dramatic developmental changes to reach mature reproductive functions. Early puberty onset is recognized as an established risk factor for all-cause mortality, hormone-related cancers, insulin resistance and obesity later in life [1, 2]. Thus, factors influencing puberty timing have been increasingly acknowledged [3, 4]. Over the last 10 years, the impact of childhood dietary phytoestrogen intake on puberty timing has been discussed. Isoflavones, the major class of dietary phytoestrogen [5], is structurally and functionally similar to endogenous oestrogen [6]. Whether isoflavone intake in childhood is implicated in the timing of puberty is currently a controversially debated issue. Support for its relevance has come from prospective studies, primarily conducted in girls, which showed that higher dietary isoflavone intake was associated with later breast development [7] or menarche [8] among German [7] and US [8] girls. In contrast, Wolff et al. [9, 10] showed that urinary isoflavone levels were not associated with breast or pubic hair development, and dietary isoflavone intake was not associated with voice break [7]. To date, the above studies were all conducted in Western countries. Since soy and its products, which are the major food sources of dietary isoflavones, are substantially consumed in developing countries, prospective investigation and understanding of the role of dietary isoflavones in puberty timing in these developing populations have important implications.

Moreover, food rich in isoflavones often contains large amounts of dietary fibre. Findings on the association of dietary fibre intake with pubertal development have been inconsistent among prospective cohorts, and evidence from non-Western populations has been lacking: high fibre consumption was associated with later menarche among Dutch [11] and Canadian [12] girls, while a null association was reported in US girls [13] and German children [7]. Therefore, the relevance of dietary isoflavones and fibre intake with puberty timing merits simultaneous evaluation. Interestingly, dietary fibre has been suggested to be associated with isoflavone bioavailability [14]. Isoflavones are transformed into aglycones with the help of gut microbes, leading to easier absorbance and higher biological activities [15]. Equol is an isoflavone bacterial metabolite and a key player in soy-related health benefits [16], and its production relies on equol-producing bacteria and is supported by fibre intake. More equol producers were reported in populations with higher dietary fibre intake, e.g., approximately 30% of omnivorous Caucasians [17], 59% of vegetarians [18] and 60.4% of Asians [19], presumably due to microbiota heterogeneity and dietary preferences for fibre subtypes favouring equol-producing bacterial growth. Nonetheless, existing studies have not considered the impacts of equol excretion or fibre subtype on pubertal development.

Over the last 40 years, a noteworthy secular trend in early puberty onset has been observed in both Chinese boys (from 16.1 years old in 1979 [20] to 14.3 years old in 2010 [21]) and girls (from 13.5 years old in 1979 [22] to 12.3 years old in 2014 [23]). Given that children with early puberty onset are associated with a spectrum of diseases leading to increasing disease burden in China [24–26], the influences of dietary isoflavones and fibre intake on puberty timing, like those investigated in our study, which include a large representative sample of Chinese children, will have important public health implications. Because the Chinese food component database is under construction, we conducted this analysis with dietary soy intake rather than calculating dietary isoflavones. Using prospective data from the Chinese Adolescent Cohort Study (CAC), we investigated the hypothesis that higher childhood dietary soy and fibre intake was associated with later puberty timing (as indicated by the early pubertal markers of age at Tanner stage 2 for breast development in girls (B2) and age at the initiation of gonadal growth in boys (G2) and the late pubertal markers of age at menarche (M) and age at voice break (VB)) and that this association could be modified both by urinary equol excretion and cereal fibre intake.

## Methods

### Study design and participants

The CAC study details have been described elsewhere [27]. Cooperative and voluntary children aged 6–8 years old in 23 selected schools have been recruited yearly since 2013. Baseline information included sociodemographic issues, dietary intake and eating behaviours, physical activity and sedentary behaviours, anthropometry and pubertal development. Follow-up data on nutrition, growth, metabolism, and health status were collected at regular intervals until the children were 15 years old: anthropometry and puberty assessments were conducted annually, and dietary intake and physical activity data were collected biennially. This study was approved by the Ethics Committee of Sichuan University, and all of the parents of the participants provided their written confirmed consent before enrolment. All examinations and questionnaires were administered with parental consent.

Between January 2013 and December 2018, 6967 children aged 6–8 years old were included at baseline. Of these children, 5439 had completed at least 2 follow-up assessments by the end of 2020. Since we were interested in the prospective relevance of diet to puberty timing, 389 children who had already reached B2/G2 at baseline were excluded from our current analysis. Among them, 141 participants with implausible energy intakes (less than or greater than age- and sex-specific cut-offs) [28] and 128 children with incomplete information on potential confounders were further excluded. In total, 4781 children (2152 girls, 2629 boys) were eligible (Additional file 1: Fig. S1), and of them, 1311 children provided first morning voided midstream urine samples.

### Nutrition assessment

Nutrition data were collected via a validated food frequency questionnaire (FFQ) by trained investigators [29]. This FFQ included 17 categories of the 53 most representative local foods or food groups among children: whole grains, refined grains, tubers, vegetables, fruits, nuts, meat, fish and shrimp, animal viscera, eggs, dairy and products, total soy (soybean and products), fried foods, sugary snacks, sugar-sweetened beverages, fruit juices and dietary supplements. The participants reported their frequency (never, daily, weekly, monthly or annually) for each item and estimated portion sizes using food models and picture aids. During the interviews, the investigators checked FFQs for potentially incorrect responses and made clarifications when necessary. Dietary intake data were converted into energy and nutrient intake data using the continuously updated in-house nutrient database based on NCCW software (version 11.0, 2014), which reflects food composition in China.

This study investigated individual mean daily intakes of total soy (soy and soy products), dietary fibre and major fibre subtypes (cereal fibre: cereals, noodle, rice, tubers, cookies and cakes; fruit fibre: fruits and its products; vegetable fibre: vegetables and its products).

### Urine analysis

Detailed instructions on collecting first morning voided midstream urine samples were carefully given to parents and children. All urine samples were stored immediately at − 20 °C before transportation and then at − 80 °C until analysis. Equol levels were determined using a previously validated gas chromatography-mass spectrometry method [7]. The detection limit was 3.8 ng/ml. All laboratory equipment was calibrated, and blinded duplicate samples were used. All of the data were double entered into the database.

### Puberty timing

According to Tanner stage standardized criteria [28], B2 and pubic hair (girls and boys) were assessed at each examination by investigators. G2 was assessed by comparative palpation with a Prader orchidometer. If the volumes of the two testes varied, the larger volume was recorded. Testicular volume less than 1 mL was recorded as 1. Moreover, children were asked whether M or VB occurred during the annual physical examination; if so, respective months and years were recorded.

### Anthropometry

An ultrasonic weight and height metre (DHM-30, Dingheng Ltd, Zhengzhou, China) was used to assess standing height to the nearest 0.1 cm and weight to the nearest 0.1 kg with the subject lightly dressed and barefoot. Triceps skinfold thicknesses and subscapular angle sites were measured on the right side to the nearest 0.1 mm using Holtain callipers (Holtain Ltd, Crymych, UK). All measurements were performed twice to calculate averages. Body mass index (BMI) sex- and age-independent BMI standard deviation scores (SDS) were calculated using Chinese reference curves [30]. Overweight was defined according to the International Obesity Task Force (IOTF) BMI cut-offs for children, which corresponds to an adult BMI of 25 kg/m^2^ [31]. The percent body fat (%BF) was calculated using Slaughter equations [32].

### Covariates

Information on the frequency, duration and type of physical activity in various settings among children was collected by a validated physical activity questionnaire with 38 items (e.g., walking, running, climbing stairs, ball games, dancing) [33]. The participants reported typical time spent on sedentary behaviours associated with television, computers, smartphones and homework.

Furthermore, parents provided information about pregnancy and infancy (i.e., children's birth weight, exclusive breastfeeding duration, timing of complementary feeding) and domestic characteristics (i.e., residency, income, family size, smoking status, parental age, occupations and education level).

### Statistical analysis

SAS® procedures (version 9.4, SAS Inc., Cary, NC, USA) and Stata 14 (Stata Corp., College Station, TX, USA) were used for data analyses. All analyses were performed with a significance level of *p*<0.05. Although there was no statistical interaction between dietary soy intake and sex, in theory, dietary oestrogen, similar to endogenous oestrogen, might differentially impact the course of puberty in girls and boys [34]. Data from girls and boys were thus analysed separately.

Since energy intake has been suggested to influence pubertal development, and energy intake is dependent on age [35], intake of total soy was expressed as age-specific residuals from the regression of soybean and its product intake on energy intake. Similarly, dietary fibre intake was expressed as age-specific residuals from the regression of fibre intake on energy intake. To examine the potential associations of total soy intake or dietary fibre intake with puberty timing, their distributions were grouped into tertiles (T1–T3).

The Kolmogorov-Smirnov and Shapiro-Wilk tests were conducted to examine the data for normality. Baseline birth weight and %BF were nonnormally distributed and presented as medians together with the interquartile rages; other continuous variables were normally distributed and presented as the means with their standard deviations (SD). Differences in anthropometric, sociodemographic and nutritional data between tertiles were analysed using an ANOVA for normally distributed continuous variables, the Kruskal-Wallis test for nonnormally distributed continuous variables, and the chi-square test for categorical variables. Statistical models and descriptive tables were stratified by sex.

Cox proportional hazard regression models were used to investigate the prospective relevance of total soy or dietary fibre (and its types) intake at baseline with age at B2/G2 or M/VB. Censoring occurred at the age of reaching B2/G2 and M/VB or age at the last follow-up if puberty events had not been reported.

In the basic models, the tertiles of total soy intake (residuals) or dietary fibre (and its types) intake (residuals) at baseline were the main independent fixed effects. These following potential confounders were considered for the Cox regression models: birth weight, age at baseline, school location, physical activity, body composition (*Z* scores of BMI, overweight (Y/N), %BF), parental/paternal/maternal educational level, family income, mother’s age at menarche, smoking status in the household, and total energy intake at baseline, as well as dietary fibre intake (residual) at baseline (in the total soy intake model) and total soy intake (residual) at baseline (in the dietary fibre intake model). In addition, we conceptualized confounders using the DAG platform [36] to validate and justify the potential confounders. Each potential confounder was initially considered separately and was included if it was associated with both the dietary index and indicators of puberty timing and if it substantially altered the estimate by more than 10% [37]. As high levels of isoflavones and dietary fibre often coexist in food, Model 2 was adjusted for parental education level, energy intake at baseline, mother’s age at menarche, and fibre intake (residuals) at baseline (in the total soy intake model) or total soy intake (residuals) at baseline (in the dietary fibre intake model). In the final model, we controlled for confounding and/or mediation by percent body fat at baseline (Model 3), because it has been proposed that body composition in childhood might be relevant to the timing of puberty [38]. Hazard ratios (HRs) and 95% confidence intervals (CIs) were estimated by comparing the 2nd and 3rd tertiles to the 1st tertile in these models. We assessed the linear trends by entering the value of dietary fibre/soy intake as a continuous variable in the above models.

To explore potential nonlinear relationships, we examined the associations (based on Model 3) of dietary soy intake and fibre intake with pubertal markers using restricted cubic spline models (four knots, according to Harrell’s recommendation [39]) among all of the participants. Four knots offer an adequate fit of the model and constitute a good compromise between flexibility and loss of precision caused by overfitting.

Moreover, we tested the potential interactions of urinary equol level (or fibre and its subtype intake) on the relationship between dietary soy intake and puberty timing. Further stratified analyses were conducted if the *p* for interaction was < 0.05.

To test the robustness of our results, we re-run our analyses using mixed model (PROC MIXED in SAS) with school clustering as a random effect, to investigate the associations of total soy intakes or fibre intakes in childhood with puberty timing.

## Results

### Characteristics

Characteristics are presented according to tertiles of soy intake (Table 1). The mean baseline age was 7.2 (0.7) years old for girls and 7.3 (0.6) years old for boys. Among girls, 1748 (81.2%) and 1162 (54.0%) reached B2 and M, respectively, soy intake varied from 0 to 69 g/day, and the highest tertile had a lower baseline %BF and higher fibre intake. In boys, 1233 (46.9%) and 829 (31.5%) reached G2 and VB, respectively, soy intake ranged from 0 to 82.6 g/day and the highest tertile had a lower baseline %BF and a higher parental education level. According to the Chinese food component database [40], the estimated isoflavone intake was approximately 65 mg/day in girls and 87 mg/day in boys, based on the mean soy intake of our sample. There were no significant differences in age, BMI SDS or %BF between eligible and excluded participants.

**Table 1:** Characteristics of participants by tertile of total soy intakes at baseline^1^

	Total soy intakes at baseline			
	T1 (0–8.2)^2^	T2 (8.5–39.6)^2^	T3 (40.1–69.0)^2^	*p*^3^
**Girls (*n*=2152)**				
*N* (%)	717 (33.3)	718 (33.4)	717 (33.3)	
Birth weight (kg)	3.1 (2.7, 3.8)	3.2 (2.9, 3.7)	3.2 (2.6, 3.8)	0.4
Age at baseline (years)	7.0 (0.8)	7.2 (0.7)	7.1 (0.8)	0.2
Age at menarche (years, *n*=1162)	12.5 (0.8)	12.8 (0.7)	13.1 (1.1)	0.03
Age at Tanner stage B2^4^ (years, *n*=1748)	9.1 (1.3)	9.3 (1.4)	9.5 (1.2)	0.02
BMI SDS at baseline (kg/m^2^)	0.1 (0.7)	0.1 (0.6)	0.2 (0.8)	0.09
Percent body fatness^5^at baseline (%)	16.7 (15.2, 19.5)	16.3 (15.0, 18.6)	15.6 (13.7, 18.5)	0.04
Overweight^6^ (*n* (%))	96 (13.4)	85 (11.8)	80 (11.2)	0.09
High physical activity (*n* (%))	190 (26.5)	202 (28.1)	198 (27.6)	0.1
**Parental data at baseline**				
High family income^7^ (*n* (%))	151 (21.1)	158 (22.0)	169 (23.6)	0.06
High paternal educational level^8^ (*n* (%))	144 (20.1)	166 (23.1)	186 (25.9)	0.05
High maternal educational level^8^ (*n* (%))	127 (17.7)	133 (18.5)	137 (19.1)	0.06
Smoking in the household (*n* (%))	471 (65.8)	408 (56.8)	345 (48.1)	0.03
Mother’s age at menarche (years)	12.1 (1.0)	12.3 (0.9)	12.4 (1.1)	0.08
**Nutritional data**^9^				
Total energy intake (kcal/day)	1594 (236)	1749 (242)	1638 (251)	0.05
Total soy (soybean and products) (g/day)	6.1 (3.9)	29.2 (8.7)	56.7 (9.8)	<0.0001
Total fibre (g/day)	10.3 (1.3)	10.5 (1.2)	10.6 (1.4)	0.06
Cereal fibre (g/day)	5.5 (1.0)	5.5 (0.9)	5.6 (1.1)	0.2
Vegetable and fruit fibre (g/day)	4.2 (1.3)	4.3 (1.1)	4.1 (1.1)	0.1
Carbohydrate (% of energy)	56.6 (5.0)	57.8 (4.8)	57.2 (4.6)	0.2
Fat (% of energy)	29.1 (4.6)	27.4 (4.2)	28.7 (4.3)	0.3
Protein (% of energy)	14.3 (2.0)	14.8 (2.1)	14.1 (1.9)	0.2
	T1 (0–3.2)^10^	T2 (4.6–48.2)^10^	T3 (49 1–82.6)^10^	*p*^3^
**Boys (*n*=2629)**				
*N* (%)	876 (33.3)	876 (33.3)	877 (33.3)	
Birth weight (kg)	3.6 (3.0, 3.9)	3.4 (2.7, 4.1)	3.4 (2.5, 4.0)	0.3
Age at baseline (years)	7.1 (0.8)	7.3 (0.6)	7.3 (0.7)	0.1
Age at voice break (years, *n*=829)	13.6 (1.3)	13.9 (1.2)	14.1 (1.2)	0.04
Age at pubertal stage G2^4^ (years, *n*=1233)	10.8 (1.3)	11.1 (1.2)	11.4 (1.2)	0.04
BMI SDS at baseline (kg/m^2^)	0.1 (0.6)	0.1 (0.8)	0.2 (0.8)	0.1
Percent body fatness^5^ at baseline (%)	13.7 (11.0, 17.4)	13.5 (10.1, 17.2)	13.5 (10.6, 17.5)	0.05
Overweight^6^ at baseline (*n* (%))	122 (13.9)	107 (12.2)	104 (11.9)	0.06
High physical activity (*n* (%))	239 (27.3)	259 (29.6)	255 (29.1)	0.07
**Parental data at baseline**				
High family income^7^ (*n* (%))	182 (20.8)	183 (20.9)	186 (21.2)	0.06
Secondary paternal educational level^8^ (*n* (%))	198 (22.6)	209 (23.9)	221 (25.2)	0.04
Secondary maternal educational level^8^ (*n* (%))	159 (18.2)	163 (18.6)	167 (19.0)	0.03
Smoking in the household (n (%))	594 (67.8)	504 (57.5)	458 (52.2)	0.04
Mother’s age at menarche (years)	12.1 (0.8)	12.3 (0.9)	12.2 (0.9)	0.06
**Nutritional data**^9^				
Total energy intake (kcal/day)	1829 (249)	1961 (225)	1790 (229)	0.1
Total soy (soybean and products) (g/day)	1.6 (1.3)	36.9 (7.6)	57.2 (8.7)	<0.0001
Total fibre (g/day)	9.8 (1.2)	10.1 (1.3)	9.9 (1.2)	0.1
Cereal fibre (g/day)	4.3 (1.1)	5.2 (1.0)	5.3 (1.1)	0.1
Vegetable and fruit fibre (g/day)	3.9 (1.2)	3.8 (1.3)	4.1 (1.2)	0.2
Carbohydrate (% of energy)	58.5 (6.3)	60.8 (6.1)	59.4 (5.9)	0.2
Fat (% of energy)	27.2 (4.3)	26.1 (4.1)	26.8 (4.2)	0.6
Protein (% of energy)	14.3 (2.1)	13.1 (2.2)	12.8 (2.1)	0.3

^1^ Values are means (SD), medians (Q1, Q3) or frequency

^2^ Values are min-max in tertiles in girls

^3^ Test for difference between the groups was performed, using ANOVA test for normal distributed continuous variables, Kruskal-Wallis test for not normally distributed continuous variables, and chi-square test for categorical variables

^4^ Tanner stage 2 for breast development (girls) or the initiation of gonadal growth (boys)

^5^ Calculated according to Slaughter equations [32]

^6^ Definition according to the International Obesity Task Force (IOTF) [31]

^7^ Average annual income of family at least ≥ 35,000 CNY (Chinese Yuan), which is a moderate level in the general population in South China [41]

^8^ School education at least 12 years

^9^ Mean values of dietary data at baseline using food frequency questionnaires

^10^ Values are min-max in tertiles in boys

### The relevance of dietary soy intake with puberty timing

In both girls and boys, higher total soy intake was correlated with later puberty timing, independent of prepubertal %BF and fibre intake (Table 2, *p* for trend ≤ 0.03): girls with high dietary total soy intake had a 12% lower risk of reaching B2 or a 13% lower risk of experiencing M than girls with low dietary total soy intake, and boys with high dietary total soy intake had a 9% lower risk of reaching G2 or a 10% lower risk of experiencing VB than boys with low dietary total soy intake. Similar results were observed in the sensitivity analysis using mixed model with school clustering as a random effect (Additional file 2: Tables S1 and S2).

**Table 2:** Associations of total soy intakes in childhood with puberty timing^1^

	Total soy intakes at baseline			
	T1 (0–8.2)^2^	T2 (8.5–39.6)^2^	T3 (40.1–69.0)^2^	*p*_trend_^3^
**Girls**				
**Age at Tanner stage B2 (*n*=2152)**				
Basic model	1	0.93 (0.86, 0.98)	0.91 (0.82, 0.97)	0.03
Model 2^4^	1	0.92 (0.84, 0.97)	0.88 (0.80, 0.97)	0.02
Model 3^5^	1	0.92 (0.84, 0.98)	0.88 (0.80, 0.96)	0.02
**Age at menarche (*n*=2152)**				
Basic model	1	0.91 (0.83, 0.98)	0.88 (0.79, 0.96)	0.03
Model 2^4^	1	0.89 (0.81, 0.97)	0.87 (0.77, 0.95)	0.01
Model 3^5^	1	0.89 (0.81, 0.96)	0.87 (0.77, 0.94)	0.01
**Boys**	T1 (0–3.2)^6^	T2 (4.6–48.2)^6^	T3 (49 1–82.6)^6^	*p*_trend_^3^
**Age at Tanner stage G2 (*n*=2629)**				
Basic model	1	0.96 (0.90, 1.01)	0.91 (0.84, 0.99)	0.04
Model 2^4^	1	0.95 (0.90, 0.99)	0.91 (0.83, 0.98)	0.03
Model 3^5^	1	0.95 (0.90, 0.99)	0.91 (0.82, 0.98)	0.03
**Age at voice break (*n*=2629)**				
Basic model	1	0.95 (0.87, 1.02)	0.92 (0.86, 1.01)	0.04
Model 2^4^	1	0.94 (0.88, 0.98)	0.90 (0.82, 0.96)	0.02
Model 3^5^	1	0.94 (0.89, 0.98)	0.90 (0.82, 0.97)	0.02

^1^ Values are models adjusted hazard ratios (95% CI), *HR* hazard ratio

^2^ Values are min-max in tertiles in girls

^3^ P for trend across tertiles were performed by including total soy intakes at baseline as continuous variables

^4^ Adjusted for parental education level, energy intake at baseline, dietary fibre intakes (residuals) at baseline and mother’s age at menarche

^5^ Additionally adjusted for percent body fat at baseline

^6^ Values are min-max in tertiles in boys

**Fig. 1:**
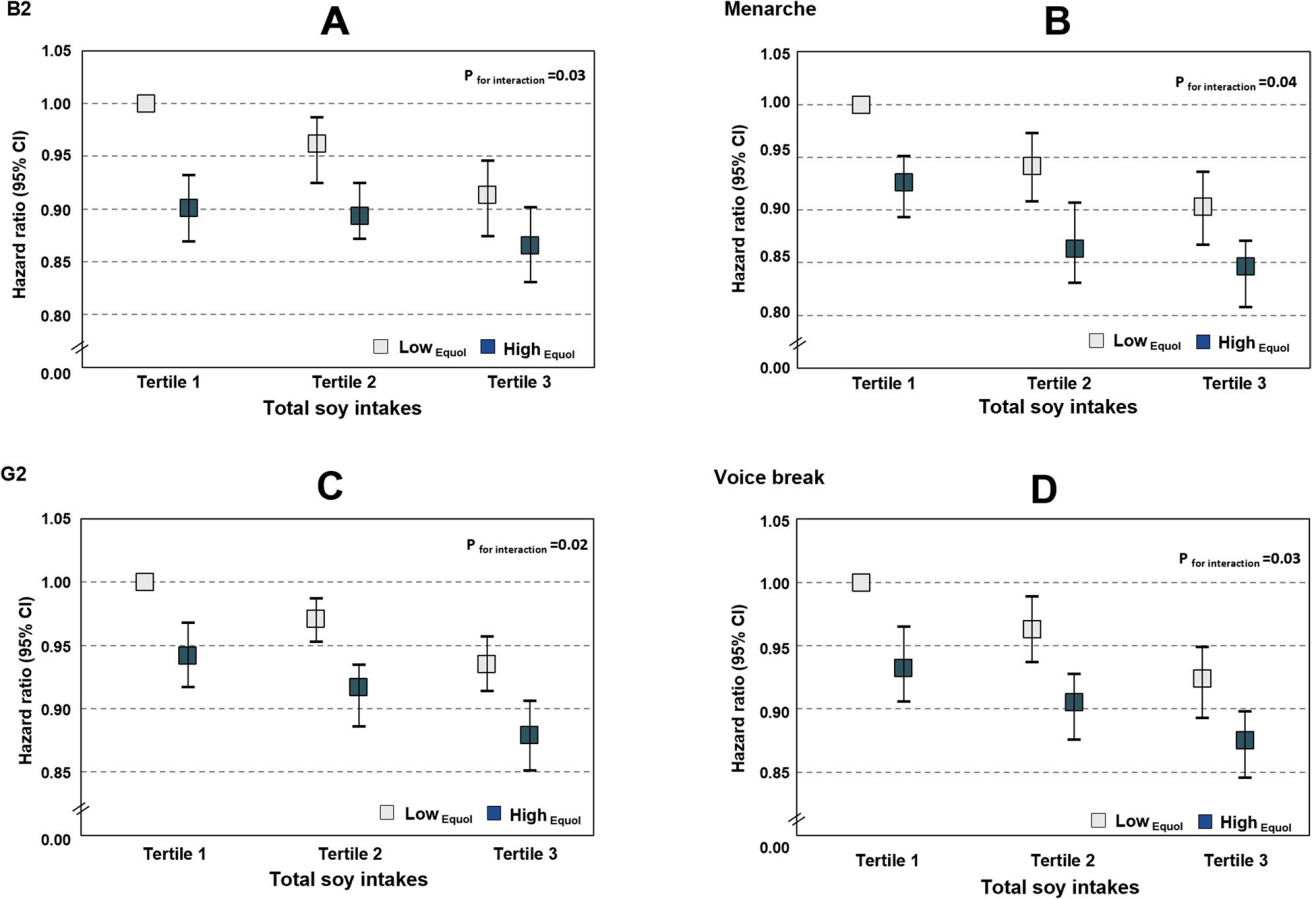
HR and 95%CI for B2 (**A**), M (**B**), G2 (**C**) and VB (**D**) stratified by urinary equol levels. Data are HR with 95% confidence intervals. Cox proportional hazard regression models were used, adjusted for parental education level, mother’s age at menarche, energy intake at baseline, dietary fibre intakes (residuals) at baseline and percent body fat at baseline, with the group of those in both lowest tertile of total soy intakes and lower equol level (< median values) serving as the reference group. *p *for interactions refers to the 2-way interactions of urinary equol level on the relations between dietary soy intake and puberty markers. **A**, **B** *N*_girl_=589. Range of total soy intakes: 1st tertile (1.5–11.3), 2nd tertile (12.1–38.9) and 3rd tertile (39.3–68.6). Participants in groups: low soy, low equol: *n*=109; low soy, high equol: *n*=103; medium soy, low equol: *n*=95; medium soy, high equol: *n*=86; high soy, low equol: *n*=108; high soy, high equol: *n*=88. **C**, **D** *N*_boy_=722. Range of soybean intakes: 1st tertile (0–4.5), 2nd tertile (5.2–46.4) and 3rd tertile (50.2–80.9). Participants in groups: low soy, low equol: *n*=146; low soy, high equol: *n*=121; medium soy, low equol: *n*=123; medium soy, high equol: *n*=98; high soy, low equol: *n*=126; high soy, high equol: *n*=108

Interestingly, these associations were modified by the urinary equol level (*p* for interaction ≤ 0.04): girls with a high urinary equol level and a high dietary total soy intake had an approximately 13% lower risk of reaching B2 or a 16% lower risk of experiencing M than those in the opposite group (Fig. 1A, B), and boys with a high urinary equol level and a high dietary total soy intake had a 12% lower risk of reaching G2 or a 13% lower risk of experiencing VB than those in the opposite group (Fig. 1C, D). Similar results were observed in the participants with urinary equol data (Additional file 2: Table S3). In addition, we found evidence of nonlinear associations (*p* value for nonlinearity < 0.005) for dietary soy intake, with strong inverse associations with pubertal markers at low intake levels, but weaker associations at moderate to higher intake levels (Additional file 1: Fig. S2: A, B, C and D).

**Fig. 2:**
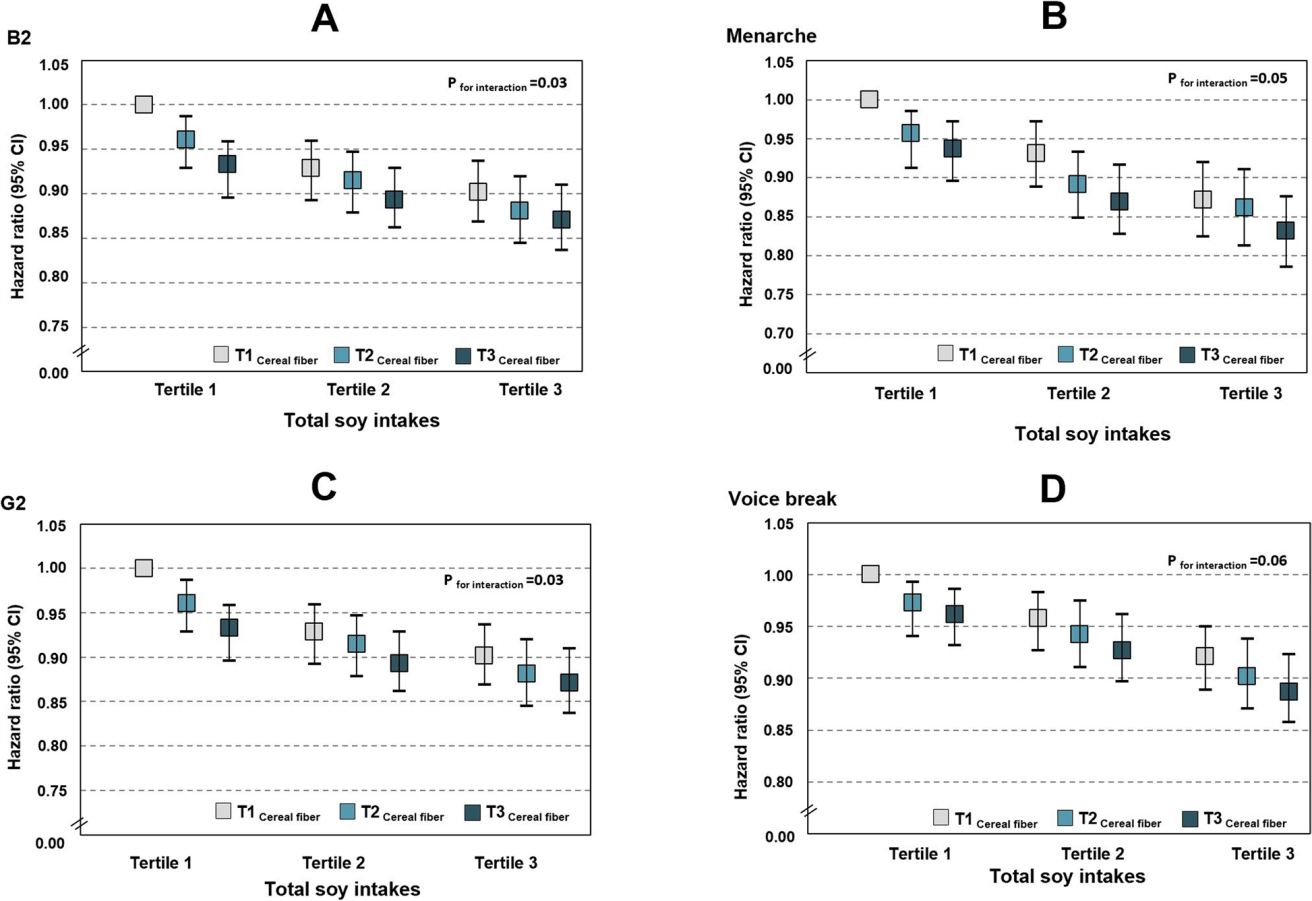
HR and 95%CI for B2 (**A**), M (**B**), G2 (**C**) and VB (**D**) stratified by cereal fibre intakes. Data are HR with 95% confidence intervals. Cox proportional hazard regression models were used, adjusted for parental education level, mother’s age at menarche, energy intake at baseline and percent body fat at baseline, with the group of those in the lowest tertile of both total soy and dietary cereal fibre intakes serving as the reference group. *p *for interactions refers to the 2-way interactions of cereal fibre intakes on the relations between dietary soy intake and puberty markers. **A**, **B** *N*_girl_=2152. Range of soybean intakes: 1st tertile (0–8.2), 2nd tertile (8.5–39.6) and 3rd tertile (40.1–69.0). Participants in groups: low soy, low cereal fibre: *n*=285; low soy, medium cereal fibre: *n*=217; low soy, high cereal fibre: *n*=222; medium soy, low cereal fibre: *n*=265; medium soy, medium cereal fibre: *n*=223; medium soy, high cereal fibre: *n*=220; high soy, low cereal fibre: *n*=309; high soy, medium cereal fibre: *n*=221; high soy, high cereal fibre: *n*=192. **C**, **D** *N*_boy_=2629. Range of soybean intakes: 1st tertile (0–3.2), 2nd tertile (4.6–48 2) and 3rd tertile (49.1–82.6). Participants in groups: low soy, low cereal fibre: *n*=350; low soy, medium cereal fibre: *n*=266; low soy, high cereal fibre: *n*=269; medium soy, low cereal fibre: *n*=320; medium soy, medium cereal fibre: *n*=272; medium soy, high cereal fibre: *n*=268; high soy, low cereal fibre: *n*=376; high soy, medium cereal fibre: *n*=274; high soy, high cereal fibre: *n*=234

Furthermore, the association of total soy intake with puberty timing was modified by the cereal fibre intake (*p* for interaction ≤ 0.06): girls with a high cereal fibre intake and a high dietary total soy intake had approximately 13% lower risk of reaching B2 or an 18% lower risk of experiencing M than those in the opposite group (Fig. 2A, B). Boys with a high cereal fibre intake and a high dietary total soy intake had an 11% lower risk of reaching G2 or a 12% lower risk of experiencing VB than those in the opposite group (Fig. 2C, D). Similar effects were not observed for vegetable and fruit fibre intake (data not shown).

### The relevance of dietary fibre intake with puberty timing

Total fibre intake was not significantly associated with puberty timing after adjusting for soy intake (Table 3, *p* for trend ≥ 0.06). Results remained unchanged in the sensitivity analysis using mixed model with school clustering as a random effect (Additional file 2: Table S4). Similar results were observed with subtype fibre intake (Additional file 2: Tables S5 and S6). Moreover, the associations between dietary fibre intake and pubertal markers appeared to be largely linear (Additional file 1: Fig. S2: E, F, G and H).

**Table 3:** Associations of total dietary fibre intakes in childhood with puberty timing ^1^

	Total dietary fibre intakes at baseline			
	T1 (2.1–6.9)^2^	T2 (7.1–9.7)^2^	T3 (9.8–14.2)^2^	*p*_trend_^3^
**Girls**				
**Age at Tanner stage B2 (*n*=2152)**				
Basic model	1	0.96 (0.90, 1.07)	0.93 (0.87, 1.09)	0.04
Model 2^4^	1	0.96 (0.86, 1.12)	0.94 (0.86, 1.11)	0.07
Model 3^5^	1	0.96 (0.86, 1.13)	0.93 (0.87, 1.11)	0.08
**Age at menarche (*n*=2152)**				
Basic model	1	0.97 (0.90, 1.07)	0.95 (0.89, 1.10)	0.03
Model 2^4^	1	0.96 (0.87, 1.10)	0.94 (0.88, 1.11)	0.06
Model 3^5^	1	0.96 (0.88, 1.12)	0.94 (0.88, 1.11)	0.06
**Boys**	T1 (1.7–5.8)^6^	T2 (6.0–8.9)^6^	T3 (9.0–13.8)^6^	*p*_trend_^3^
**Age at Tanner stage G2 (*n*=2629)**				
Basic model	1	0.95(0.90, 1.10)	0.94 (0.88, 1.12)	0.06
Model 2^4^	1	0.94 (0.86, 1.13)	0.94 (0.86, 1.12)	0.08
Model 3^5^	1	0.94 (0.87, 1.13)	0.93 (0.87, 1.10)	0.09
**Age at voice break (*n*=2629)**				
Basic model	1	0.94 (0.87, 1.09)	0.93 (0.86, 1.11)	0.05
Model 2^4^	1	0.94 (0.86, 1.10)	0.94 (0.87, 1.11)	0.07
Model 3^5^	1	0.94 (0.88, 1.11)	0.94 (0.87, 1.10)	0.08

^1^ Values are models adjusted hazard ratios (95% CI), *HR* hazard ratio

^2^ Values are min-max in tertiles in girls

^3^ P for trend across tertiles were performed by including dietary fibre intakes at baseline as continuous variables

^4^ Adjusted for parental education level, energy intake at baseline, total soy intakes (residuals) at baseline and mother’s age at menarche

^5^ Additionally adjusted for percent body fat at baseline

^6^ Values are min-max in tertiles in boys

## Discussion

In the present analysis, girls and boys with higher total soy intake reached puberty later than children with lower soy intake, independent of prepubertal body fat and fibre consumption. These prospective associations were particularly pronounced among those with a higher urinary equol level or those with a high cereal fibre intake.

Puberty timing is a developmental milestone for children. Prospective cohort studies of soy or isoflavone intake and puberty timing have been limited. Our findings are in line with those from 230 US girls with higher phytoestrogen intake and delayed age at menarche [8] and from 119 German girls from the Dortmund Nutritional and Anthropometric Longitudinally Designed (DONALD) study with higher isoflavone intake and later breast development [7]. However, the associations between soy or isoflavone intake and age at menarche were not consistent between girls from our study and those in the DONALD study. This inconsistency might partly be due to the small sample size, population bias toward high socioeconomic status, and lack of control over potential maternal genetic influences in the DONALD study. In addition, we speculate that the average level of individual isoflavone intake might also play a role. In our study, the estimated median value of soy isoflavone intake in girls was higher than that in Western populations (approximately 65 mg/day vs. 0.04–19.2 mg/day [7, 42]). Therefore, we are led to believe that the association between isoflavone intake and age at menarche in German girls might be obscured by a low isoflavone intake level. Similarly, the relevance between isoflavone intake and puberty timing was absent in 108 boys from the DONALD Study [7]. However, we have evidently observed that higher soy intake was correlated with later age at gonadal growth and voice break in Chinese boys, possibly because the estimated isoflavone intake level in boys from our study was markedly higher than the reported isoflavone intake level in boys from the DONALD study (approximately 87 mg/day vs. 0.8–62.7 mg/day [7, 43]).

Although the mechanism underlying the association between soy intake (isoflavone) and puberty timing remains unknown, we speculate that possible explanations are: isoflavone is structurally and functionally similar to endogenous oestrogen [6] and could inhibit the activity of aromatase, the rate-limiting enzyme of oestrogen biosynthesis [44], therefore possibly affecting endocrine homeostasis and further pubertal development. Conversely, isoflavones have been reported to directly bind to and influence the expression of oestrogen receptors (ERs) [45] and impact the hypothalamus-pituitary-gonadal (HPG) axis [46]. Menarche is a late pubertal stage primarily governed by the HPG axis, whereas breast development represents an early pubertal stage influenced by both the HPG axis and peripheral factors, including oestrogen-related enzymes and ERs on gonadal cells [47]. Therefore, we speculate that low levels of isoflavone intake is able to influence breast development, which was observed in girls from the DONALD study [7], while the relevance between isoflavone intake and menarche was absent because a higher isoflavone concentration might be needed to influence the HPG axis. However, further investigations of the HPG axis and peripheral pubertal development are needed to elucidate possible underlying mechanisms.

Food rich in isoflavones typically contains appreciable amounts of dietary fibre. The present analysis demonstrated that dietary fibre and its subtypes were not independently associated with puberty timing. Our data further suggested that the associations between total soy intake and pubertal markers were more pronounced in children with higher cereal fibre intake. In our analysis, cereal fibre was defined as fibre from cereals, noodles, rice, tubers, cookies and cakes, representing a major group of influential nutrients known as microbiota-accessible carbohydrates (MACs). MACs vary distinctly in Western and traditional fibre-rich diets, leading to changes in gut microbiota, microbial functionality and bacteria-host interactions [48]. Cereal fibre intake was not independently related to puberty timing in our participants; however, it was intriguing to observe a modified effect of dietary cereal fibre on soy-puberty associations. One possible explanation is that high cereal fibre intake, hence elevated MAC levels, facilitates the establishment of a “healthy” gut microbiota favouring an isoflavone-friendly microbial ecosystem in which soy is efficiently digested, absorbed and utilized [48].

Gut microbes play a critical role in transforming glucose-conjugated soy isoflavones into aglycones, which are easier to absorb and retain higher biological activities than the glycosylated parent compounds [15]. Additionally, equol production occurs in the intestine via bacterial reductases from a number of gut microbes, and the list of equol-producing bacteria is constantly expanding with newly characterized members [16]. More equol producers have been reported in populations with higher fibre intake [17–19]. Since equol is generally considered to be the main conductor for soy-related health benefits [16], the relationship between equol and puberty onset is of particular interest. However, observational data on this matter are scarce. In this study, associations of total soy intake with puberty timing were more prominent in children with higher urinary equol levels, which might indicate that a pro-equol-production gut microbiota might reinforce the impact of soy consumption on puberty timing. Combined with the findings on cereal fibre intake, we believe that a higher intake of foods rich in MACs might build an optimal intestinal microflora, likely indicated by urinary equol levels, to efficiently deploy the puberty-influential effects of soy isoflavones.

Our study has several strengths. In contrast to most studies which focused only on girls, we observed both girls and boys. Our participants and their parents/family were representative of the general population in age, economic and educational status according to regional statistical books [28]. The prospective nature and repeated detailed measurements of anthropometric, pubertal and dietary data in participants, in conjunction with the ability to adjust for a number of major potential confounders both in children and in their parents, represent substantial strengths. Notably, prepubertal body fatness was considered in our analysis because childhood body composition might potentially influence puberty onset [49].

One of our study’s limitations is that the observational design of our study could not establish causal relationships, but possible factors have been provided for further causal investigation. Although we considered possible confounders in our statistical adjustment, there might be residual confounders that we could not capture. Third, given that our study interest was dietary fibre/soy intake at baseline, we were unable to detect eating behaviour changes during the study follow-ups, which might confound these associations. In addition, because soy and its products are widely and substantially consumed in Asian diets, isoflavone intake among Asians is considerably higher than that in Western populations [15], so that generalization of our findings might be limited in low-soy-consumption populations. Furthermore, urinary equol level might merely represent the quantity of excreted equol in the urine, rather than one’s ability to produce or utilize equol. The soy challenging test could provide valuable information about equol-production status in future cohorts.

## Conclusions

In conclusion, higher childhood soy intake is prospectively associated with later puberty timing in both Chinese girls and boys, independent of prepubertal body fatness, and is particularly pronounced among individuals with a higher urinary equol level.

## Supplementary Information

**Additional file 1: Figure S1.** Flowchart for the study sample. **Figure S2.** Shape of the associations between total soy intakes and fibre intakes in childhood with puberty timing. In the analyses, 2152 girls and 2629 boys were included. B2, Tanner stage 2 for breast development; G2, the initiation of gonadal growth.

**Additional file 2: Table S1.** Associations of total soy intakes in childhood with puberty timing using mixed model. **Table S2.** Associations of total soy intakes in childhood with puberty timing among participants with urinary equol data using mixed model. **Table S3.** Associations of total soy intakes in childhood with puberty timing among participants with urinary equol data. **Table S4.** Associations of total dietary fibre intakes in childhood with puberty timing using mixed model. **Table S5.** Associations of cereal fibre intakes in childhood with puberty timing. **Table S6.** Associations of vegetable and fruit fibre intakes in childhood with puberty timing.

## Data Availability

The datasets supporting the conclusions of this article are included within the article and its supplementary files.

## Abbreviations

CAC: The Chinese Adolescent Cohort Study
B2/G2: Age at Tanner stage 2 for breast development or age at the initiation of gonadal growth
M/VB: Age at menarche or voice break
FFQ: Food frequency questionnaire
%BF: Percent body fat
BMI: Body mass index
HR: Hazard ratio
CI: Confidential interval
HPG: Hypothalamus-pituitary-gonadal
ERs: Oestrogen receptors
MACs: Microbiota-accessible carbohydrates
