# Supplementary material for: Prospective association of dietary soy and fibre intake with puberty timing: a cohort study among Chinese children
Source: BMC Med. 2022 Apr 4;20:145. doi: 10.1186/s12916-022-02320-5 (PMC8978387; doi:10.1186/s12916-022-02320-5)
Supplement: Supplementary file 1 — Additional file 1: Figure S1. Flowchart for the study sample. Figure S2. Shape of the associations between total soy intakes and fibre intakes in childhood with puberty timing. In the analyses, 2152 girls and 2629 boys were included. B2, Tanner stage 2 for breast development; G2, the initiation of gonadal growth. [file 12916_2022_2320_MOESM1_ESM.pdf]

Figure S1. Flowchart for the study sample.

Age at tanner stage 2 for breast development (B2) and age at menarche (M) for girls, and age at tanner stage 2 for the initiation of gonadal growth (G2) and age at voice break (VB) for boys were determined.

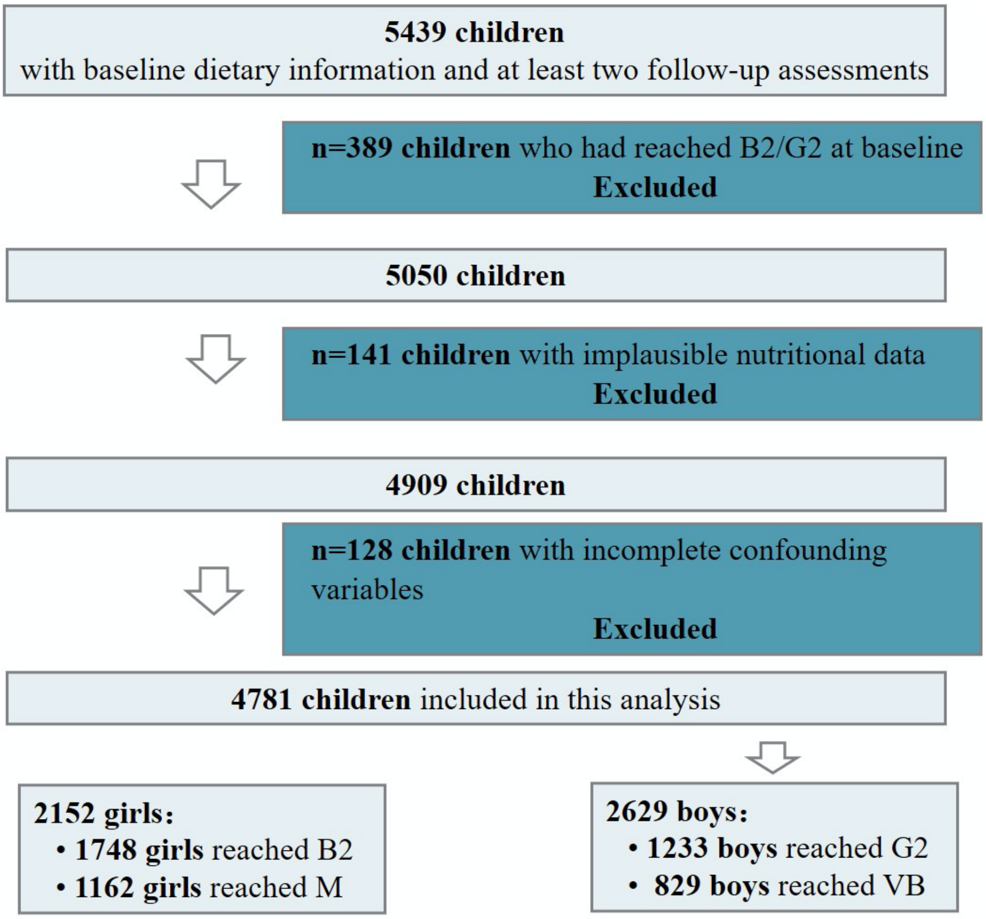

Figure S2. Shape of the association between total soy intakes and fibre intakes in childhood with puberty timing. In the analyses, 2152 girls and 2629 boys were included. B2, Tanner stage 2 for breast development; G2, the initiation of gonadal growth.

Restricted cubic spline models with 4 knots were used to estimate the association between total soy intakes and fibre intakes in childhood with B2, menarche, G2 and voice break, adjusting for parental education level, energy intake at baseline, dietary fibre intakes (residuals) at baseline and mother's age at menarche, percent body fat at baseline. For total soy intakes, we found evidence of a non-linear association (P value for non-linearity <0.005)

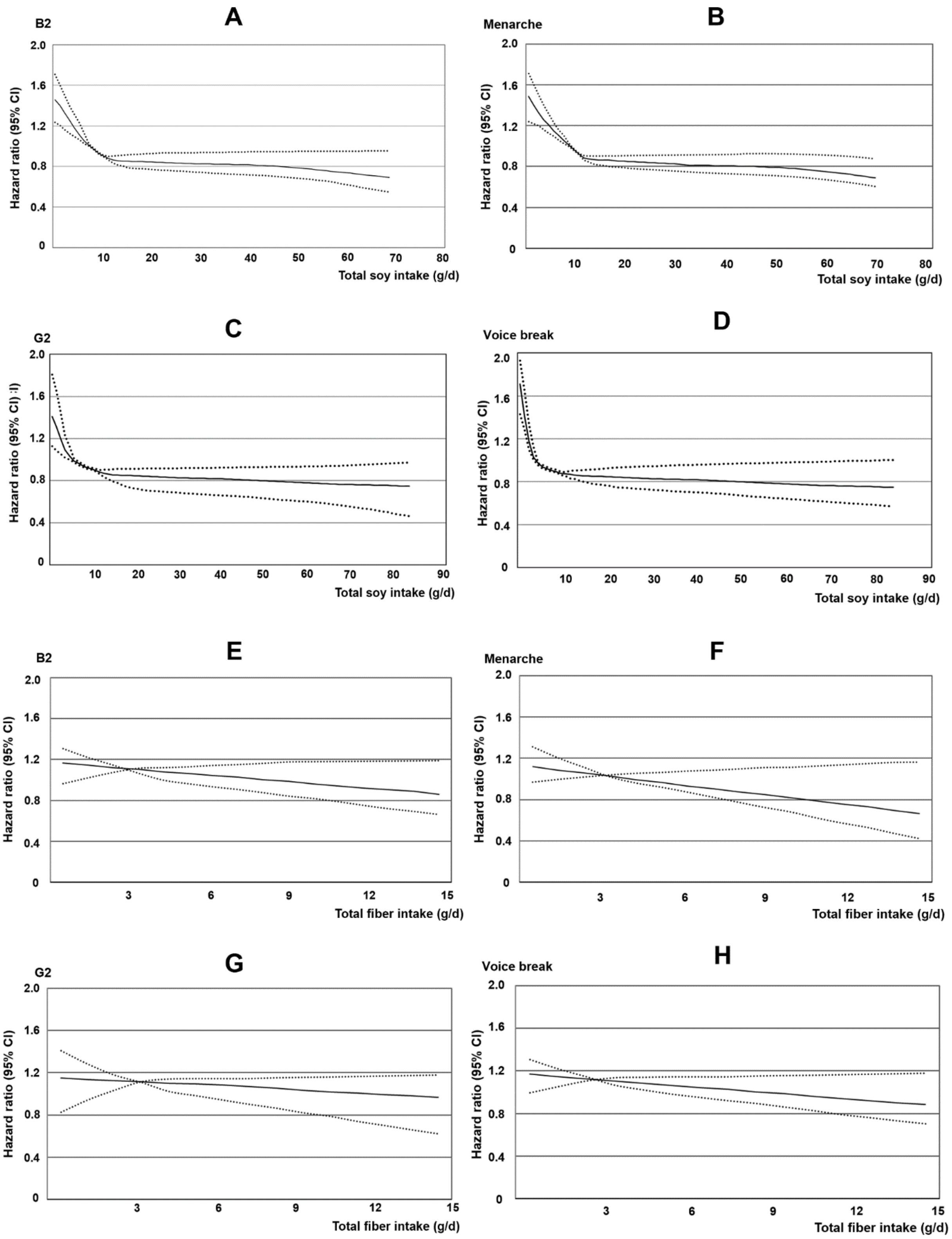
A
B2
Hazard ratio (95% CI)
Total soy intake (g/d)
B
Menarche
Hazard ratio (95% CI)
Total soy intake (g/d)
C
G2
Hazard ratio (95% CI)
Total soy intake (g/d)
D
Voice break
Hazard ratio (95% CI)
Total soy intake (g/d)
E
B2
Hazard ratio (95% CI)
Total fiber intake (g/d)
F
Menarche
Hazard ratio (95% CI)
Total fiber intake (g/d)
G
G2
Hazard ratio (95% CI)
Total fiber intake (g/d)
H
Voice break
Hazard ratio (95% CI)
Total fiber intake (g/d)
