# Supplementary material for: Prospective association of dietary soy and fibre intake with puberty timing: a cohort study among Chinese children
Source: BMC Med. 2022 Apr 4;20:145. doi: 10.1186/s12916-022-02320-5 (PMC8978387; doi:10.1186/s12916-022-02320-5)
Supplement: Supplementary file 2 — Additional file 2: Table S1. Associations of total soy intakes in childhood with puberty timing using mixed model. Table S2. Associations of total soy intakes in childhood with puberty timing among participants with urinary equol data using mixed model. Table S3. Associations of total soy intakes in childhood with puberty timing among participants with urinary equol data. Table S4. Associations of total dietary fibre intakes in childhood with puberty timing using mixed model. Table S5. Associations of cereal fibre intakes in childhood with puberty timing. Table S6. Associations of vegetable and fruit fibre intakes in childhood with puberty timing. [file 12916_2022_2320_MOESM2_ESM.docx]

**Table S1. Associations of total soy intakes in childhood with puberty timing using mixed model^1^**

|  | **Total soy intakes at baseline** | | |  |  |
| --- | --- | --- | --- | --- | --- |
|  | T1  (0-8.2)^2^ | T2  (8.5-39.6)^2^ | T3  (40.1-69.0)^2^ | *p* | AIC^3^ |
| **Girls** |  |  |  |  |  |
| **Age at Tanner stage B2 (n=2152)** | |  |  |  |  |
| Basic model: | 1 | 0.92 (0.80, 0.97) | 0.89 (0.81, 0.96) | 0.04 | 3792 |
| Model 2^4^: | 1 | 0.91 (0.83, 0.96) | 0.87 (0.78, 0.95) | 0.03 | 3672 |
| Model 3^5^: | 1 | 0.91 (0.83, 0.96) | 0.87 (0.78, 0.96) | 0.02 | 3626 |
| **Age at menarche (n=2152)** | |  |  |  |  |
| Basic model: | 1 | 0.90 (0.82, 0.96) | 0.86 (0.75, 0.96) | 0.03 | 4296 |
| Model 2^4^: | 1 | 0.87 (0.81, 0.94) | 0.84 (0.73, 0.93) | 0.01 | 4189 |
| Model 3^5^: | 1 | 0.87 (0.80, 0.93) | 0.84 (0.72, 0.93) | 0.01 | 4165 |
|  |  |  |  |  |  |
| **Boys** | T1  (0-3.2)^6^ | T2  (4.6-48.2)^6^ | T3  (49 1-82.6)^6^ | *p* | AIC^3^ |
| **Age at Tanner stage G2 (n=2629)** | |  |  |  |  |
| Basic model: | 1 | 0.95 (0.89, 0.99) | 0.91 (0.87, 0.97) | 0.03 | 3878 |
| Model 2^4^: | 1 | 0.93 (0.88, 0.98) | 0.90 (0.85, 0.96) | 0.02 | 3762 |
| Model 3^5^: | 1 | 0.93 (0.87, 0.98) | 0.90 (0.85, 0.96) | 0.02 | 3731 |
| **Age at voice break (n=2629)** | |  |  |  |  |
| Basic model: | 1 | 0.94 (0.86, 0.98) | 0.91 (0.83, 1.02) | 0.03 | 4573 |
| Model 2^4^: | 1 | 0.92 (0.88, 0.97) | 0.89 (0.84, 0.95) | 0.01 | 4432 |
| Model 3^5^: | 1 | 0.92 (0.87, 0.97) | 0.89 (0.83, 0.96) | 0.01 | 4415 |

^1^ Mixed model contain a random statement for school clustering with an unstructured covariance

^2^ Values are min-max in tertiles in girls

^3^ AIC: Akaike’s Information Criterion (smaller is better)

^4^ Adjusted for parental education level, energy intake at baseline, dietary fibre intakes (residuals) at baseline and mother’s age at menarche

^5^ Additionally adjusted for percent body fat at baseline

^6^ Values are min-max in tertiles in boys

**Table S2. Associations of total soy intakes in childhood with puberty timing among participants with urinary equol data using mixed model^1^**

|  | **Total soy intakes at baseline** | | |  |  |  |
| --- | --- | --- | --- | --- | --- | --- |
|  | T1  (1.5-11.3)^2^ | T2  (12.1-38.9)^2^ | T3  (39.3-68.6)^2^ | *p* | AIC^3^ |  |
| **Girls** |  |  |  |  |  |  |
| **Age at Tanner stage B2 (n=589)** | |  |  |  |  |  |
| Basic model: | 1 | 0.91 (0.81, 0.97) | 0.88 (0.80, 0.95) | 0.03 | 3712 |  |
| Model 2^4^: | 1 | 0.90 (0.82, 0.96) | 0.86 (0.79, 0.96) | 0.03 | 3626 |  |
| Model 3^5^: | 1 | 0.90 (0.83, 0.95) | 0.87 (0.79, 0.95) | 0.02 | 3591 |  |
| **Age at menarche (n=589)** | |  |  |  |  |  |
| Basic model: | 1 | 0.91 (0.83, 0.98) | 0.87 (0.79, 0.95) | 0.03 | 4212 |  |
| Model 2^4^: | 1 | 0.88 (0.82, 0.95) | 0.86 (0.77, 0.94) | 0.02 | 4169 |  |
| Model 3^5^: | 1 | 0.88 (0.81, 0.94) | 0.85 (0.75, 0.94) | 0.02 | 4123 |  |
|  |  |  |  |  |  |  |
| **Boys** | T1  (0-4.5)^6^ | T2  (5.2-46.4)^6^ | T3  (50.2-80.9)^6^ | *p* | AIC^3^ |  |
| **Age at Tanner stage G2 (n=722)** | |  |  |  |  |  |
| Basic model: | 1 | 0.94 (0.89, 0.97) | 0.90 (0.85, 0.96) | 0.04 | 3801 |  |
| Model 2^4^: | 1 | 0.92 (0.86, 0.98) | 0.89 (0.83, 0.95) | 0.03 | 3709 |  |
| Model 3^5^: | 1 | 0.92 (0.87, 0.97) | 0.88 (0.83, 0.94) | 0.03 | 3672 |  |
| **Age at voice break (n=722)** | |  |  |  |  |  |
| Basic model: | 1 | 0.93 (0.87, 0.98) | 0.89 (0.85, 0.99) | 0.03 | 4511 |  |
| Model 2^4^: | 1 | 0.91 (0.89, 0.98) | 0.87 (0.82, 0.96) | 0.02 | 4427 |  |
| Model 3^5^: | 1 | 0.92 (0.89, 0.97) | 0.87 (0.82, 0.95) | 0.02 | 4385 |  |

^1^ Mixed model contain a random statement for school clustering with an unstructured covariance

^2^ Values are min-max in tertiles in girls

^3^ AIC: Akaike’s Information Criterion (smaller is better)

^4^ Adjusted for parental education level, energy intake at baseline, dietary fibre intakes (residuals) at baseline and mother’s age at menarche

^5^ Additionally adjusted for percent body fat at baseline

^6^ Values are min-max in tertiles in boys

**Table S3. Associations of total soy intakes in childhood with puberty timing^1^ among participants with urinary equol data**

|  | **Total soy intakes at baseline** | | |  |
| --- | --- | --- | --- | --- |
|  | T1  (1.5-11.3)^2^ | T2  (12.1-38.9)^2^ | T3  (39.3-68.6)^2^ | *p_trend_* ^3^ |
| **Girls** |  |  |  |  |
| **Age at Tanner stage B2 (n=589)** | |  |  |  |
| Basic model: | 1 | 0.91 (0.85, 0.97) | 0.90 (0.81, 0.98) | 0.02 |
| Model 2^4^: | 1 | 0.92 (0.85, 0.98) | 0.88 (0.77, 0.96) | 0.02 |
| Model 3^5^: | 1 | 0.91 (0.84, 0.96) | 0.87 (0.78, 0.96) | 0.01 |
| **Age at menarche (n=589)** | |  |  |  |
| Basic model: | 1 | 0.90 (0.82, 0.96) | 0.88 (0.78, 0.95) | 0.02 |
| Model 2^4^: | 1 | 0.88 (0.81, 0.96) | 0.87 (0.76, 0.95) | 0.01 |
| Model 3^5^: | 1 | 0.88 (0.79, 0.95) | 0.87 (0.76, 0.94) | 0.01 |
|  |  |  |  |  |
| **Boys** | T1  (0-4.5)^6^ | T2  (5.2-46.4)^6^ | T3  (50.2-80.9)^6^ | *p_trend_* ^3^ |
| **Age at Tanner stage G2 (n=722)** | |  |  |  |
| Basic model: | 1 | 0.95 (0.91, 0.99) | 0.90 (0.85, 0.97) | 0.04 |
| Model 2^4^: | 1 | 0.95 (0.89, 0.98) | 0.91 (0.84, 0.98) | 0.03 |
| Model 3^5^: | 1 | 0.94 (0.90, 0.97) | 0.90 (0.83, 0.96) | 0.03 |
| **Age at voice break (n=722)** | |  |  |  |
| Basic model: | 1 | 0.94 (0.87, 1.01) | 0.91 (0.85, 0.99) | 0.03 |
| Model 2^4^: | 1 | 0.94 (0.88, 0.99) | 0.90 (0.83, 0.97) | 0.02 |
| Model 3^5^: | 1 | 0.93 (0.87, 0.98) | 0.91 (0.83, 0.98) | 0.02 |

^1^ Values are models adjusted hazard ratios (95% CI), HR= hazard ratio

^2^ Values are min-max in tertiles in girls

^3^ P for trend across tertiles were performed by including total soy intakes at baseline as continuous variables

^4^ Adjusted for parental education level, energy intake at baseline, dietary fibre intakes (residuals) at baseline and mother’s age at menarche

^5^ Additionally adjusted for percent body fat at baseline

^6^ Values are min-max in tertiles in boys

**Table S4. Associations of total dietary fibre intakes in childhood with puberty timing using mixed model ^1^**

|  | **Total dietary fibre intakes at baseline** | | |  |  |  |
| --- | --- | --- | --- | --- | --- | --- |
|  | T1  (2.1-6.9)^2^ | T2  (7.1-9.7)^2^ | T3  (9.8-14.2)^2^ | *p* | AIC^3^ |  |
| **Girls** |  |  |  |  |  |  |
| **Age at Tanner stage B2 (n=2152)** | |  |  |  |  |  |
| Basic model: | 1 | 0.95 (0.89, 1.05) | 0.93 (0.85, 1.06) | 0.05 | 3805 |  |
| Model 2^4^: | 1 | 0.94 (0.85, 1.07) | 0.92 (0.85, 1.09) | 0.07 | 3718 |  |
| Model 3^5^: | 1 | 0.94 (0.85, 1.07) | 0.92 (0.84, 1.10) | 0.07 | 3651 |  |
| **Age at menarche (n=2152)** | |  |  |  |  |  |
| Basic model: | 1 | 0.96 (0.88, 1.02) | 0.93 (0.89, 0.98) | 0.04 | 4137 |  |
| Model 2^4^: | 1 | 0.97 (0.86, 1.06) | 0.92 (0.86, 1.01) | 0.06 | 4021 |  |
| Model 3^5^: | 1 | 0.97 (0.86, 1.07) | 0.92 (0.86, 0.99) | 0.06 | 3986 |  |
|  |  |  |  |  |  |  |
| **Boys** | T1  (1.7-5.8)^6^ | T2  (6.0-8.9)^6^ | T3  (9.0-13.8)^6^ | *p* | AIC^3^ |  |
| **Age at Tanner stage G2 (n=2629)** | | |  |  |  |  |
| Basic model: | 1 | 0.96 (0.89, 1.02) | 0.93 (0.86, 1.01) | 0.05 | 3902 |  |
| Model 2^4^: | 1 | 0.95 (0.87, 1.05) | 0.91 (0.82, 1.03) | 0.08 | 3867 |  |
| Model 3^5^: | 1 | 0.95 (0.87, 1.06) | 0.91 (0.82, 1.05) | 0.08 | 3812 |  |
| **Age at voice break (n=2629)** | |  |  |  |  |  |
| Basic model: | 1 | 0.94 (0.83, 1.06) | 0.92 (0.83, 1.01) | 0.05 | 4315 |  |
| Model 2^4^: | 1 | 0.93 (0.86, 1.03) | 0.90 (0.78, 1.08) | 0.07 | 4236 |  |
| Model 3^5^: | 1 | 0.93 (0.85, 1.05) | 0.90 (0.77, 1.09) | 0.07 | 4201 |  |

^1^ Mixed model contain a random statement for school clustering with an unstructured covariance

^2^ Values are min-max in tertiles in girls

^3^ AIC: Akaike’s Information Criterion (smaller is better)

^4^ Adjusted for parental education level, energy intake at baseline, dietary fibre intakes (residuals) at baseline and mother’s age at menarche

^5^ Additionally adjusted for percent body fat at baseline

^6^ Values are min-max in tertiles in boys

**Table S5. Associations^1^ of cereal fibre intakes in childhood with puberty timing.**

|  | **Cereal fibre intakes at baseline** | | |  |
| --- | --- | --- | --- | --- |
|  | T1  (0.9-2.9)^2^ | T2  (3.1-4.6)^2^ | T3  (4.7-7.1)^2^ | *p_trend_* ^3^ |
| **Girls** |  |  |  |  |
| **Age at Tanner stage B2 (n=2152)** | |  |  |  |
| Basic model: | 1 | 0.97 (0.87, 1.13) | 0.96 (0.85, 1.15) | 0.1 |
| Model 2^4^: | 1 | 0.96 (0.85, 1.14) | 0.97 (0.86, 1.13) | 0.1 |
| Model 3^5^: | 1 | 0.96 (0.85, 1.13) | 0.96 (0.84, 1.13) | 0.2 |
| **Age at menarche (n=2152)** | |  |  |  |
| Basic model: | 1 | 0.93 (0.84, 1.15) | 0.96 (0.88, 1.16) | 0.08 |
| Model 2^4^: | 1 | 0.93 (0.85, 1.14) | 0.95 (0.88, 1.15) | 0.2 |
| Model 3^5^: | 1 | 0.94 (0.86, 1.15) | 0.95 (0.87, 1.15) | 0.2 |
|  |  |  |  |  |
| **Boys** | T1  (1.1-3.6)^6^ | T2  (3.7-5.0)^6^ | T3  (5.1-7.4)^6^ | *p_trend_* ^3^ |
| **Age at Tanner stage G2 (n=2629)** | |  |  |  |
| Basic model: | 1 | 0.99 (0.90, 1.16) | 1.02 (0.92, 1.17) | 0.09 |
| Model 2^4^: | 1 | 1.03 (0.92, 1.19) | 1.03 (0.93, 1.21) | 0.1 |
| Model 3^5^: | 1 | 1.01 (0.91, 1.15) | 1.03 (0.92, 1.19) | 0.2 |
| **Age at voice break (n=2629)** | |  |  |  |
| Basic model: | 1 | 1.02 (0.92, 1.18) | 1.04 (0.95, 1.21) | 0.07 |
| Model 2^4^: | 1 | 1.03 (0.92, 1.19) | 1.03 (0.92, 1.19) | 0.1 |
| Model 3^5^: | 1 | 1.02 (0.91, 1.17) | 1.03 (0.93, 1.19) | 0.2 |

^1^ Values are models adjusted hazard ratios (95% CI), HR= hazard ratio

^2^ Values are min-max in tertiles in girls

^3^ P for trend across tertiles were performed by including cereal fibre intakes at baseline as continuous variables

^4^ Adjusted for parental education level, energy intake at baseline, dietary soy intakes (residuals) at baseline and mother’s age at menarche

^5^ Additionally adjusted for percent body fat at baseline

^6^ Values are min-max in tertiles in boys

**Table S6. Associations^1^ of vegetable and fruit fibre intakes in childhood with puberty timing.**

|  | **Vegetable and fruit fibre intakes at baseline** | | |  |
| --- | --- | --- | --- | --- |
|  | T1  (0.8-2.8)^2^ | T2  (2.9-3.9)^2^ | T3  (4.0-5.8)^2^ | *p_trend_* ^3^ |
| **Girls** |  |  |  |  |
| **Age at Tanner stage B2 (n=2152)** | |  |  |  |
| Basic model: | 1 | 0.99 (0.91, 1.15) | 0.99 (0.89, 1.17) | 0.2 |
| Model 2^4^: | 1 | 0.97 (0.89, 1.14) | 0.98 (0.90, 1.16) | 0.3 |
| Model 3^5^: | 1 | 0.97 (0.90, 1.13) | 0.98 (0.90, 1.17) | 0.3 |
| **Age at menarche (n=2152)** | |  |  |  |
| Basic model: | 1 | 0.98 (0.91, 1.12) | 0.97 (0.89, 1.13) | 0.1 |
| Model 2^4^: | 1 | 0.98 (0.89, 1.09) | 0.96 (0.90, 1.12) | 0.1 |
| Model 3^5^: | 1 | 0.97 (0.89, 1.09) | 0.96 (0.89, 1.12) | 0.2 |
|  |  |  |  |  |
| **Boys** | T1  (0.7-2.6)^6^ | T2  (2.7-3.6)^6^ | T3  (3.7-5.3)^6^ | *p_trend_* ^3^ |
| **Age at Tanner stage G2 (n=2629)** | |  |  |  |
| Basic model: | 1 | 1.03 (0.90, 1.15) | 1.02 (0.91, 1.15) | 0.2 |
| Model 2^4^: | 1 | 1.03 (0.91, 1.14) | 1.02 (0.90, 1.16) | 0.3 |
| Model 3^5^: | 1 | 1.02 (0.90, 1.13) | 1.01 (0.91, 1.15) | 0.3 |
| **Age at voice break (n=2629)** | |  |  |  |
| Basic model: | 1 | 1.04 (0.90, 1.19) | 1.02 (0.92, 1.18) | 0.1 |
| Model 2^4^: | 1 | 1.04 (0.91, 1.18) | 1.03 (0.92, 1.18) | 0.2 |
| Model 3^5^: | 1 | 1.03 (0.90, 1.18) | 1.02 (0.91, 1.17) | 0.2 |

^1^ Values are models adjusted hazard ratios (95% CI), HR= hazard ratio

^2^ Values are min-max in tertiles in girls

^3^ P for trend across tertiles included vegetable and fruit fibre intakes at baseline as continuous variables

^4^ Adjusted for parental education level, energy intake at baseline, dietary soy intakes (residuals) at baseline and mother’s age at menarche

^5^ Additionally adjusted for percent body fat at baseline

^6^ Values are min-max in tertiles in boys
